# Pancreatic fat deposition is increased and related to beta-cell function in women with familial partial lipodystrophy

**DOI:** 10.1186/s13098-018-0375-9

**Published:** 2018-09-26

**Authors:** Amelio F. Godoy-Matos, Cynthia M. Valerio, Rodrigo O. Moreira, Denise P. Momesso, Leonardo K. Bittencourt

**Affiliations:** 1 Serviço de Metabologia, Instituto Estadual de Diabetes e Endocrinologia (IEDE), Rua Visconde Silva, 52/1135 Botafogo, Rio de Janeiro, 22271-090 Brazil; 2 Departamento de Radiologia, Universidade Federal Fluminense-Section Head, Abdominal and Pelvic Imaging-CDPI Clinics, DASA Company, Rio de Janeiro, Brazil

**Keywords:** Lipodystrophy, Pancreas, Ectopic fat, Beta cell function

## Abstract

**Background:**

To study pancreatic fat deposition and beta-cell function in familial partial lipodystrophy (FPLD) patients.

**Methods:**

In a cross-sectional study, eleven patients with FPLD, and eight healthy volunteers were matched for age and body mass index and studied at a referral center. Body composition was assessed using dual-energy X-ray absorptiometry and the Dixon method of magnetic resonance imaging was used to quantify pancreatic and liver fat. Fasting plasma glucose, insulin, leptin, lipids and homeostasis model assessment of insulin resistance values were measured, and an oral glucose tolerance test was performed. The insulinogenic index, Matsuda insulin sensitivity index and beta-cell disposition index were calculated.

**Results:**

The FPLD group presented a higher waist-to-hip ratio and fat mass ratio and lower total, truncal and lower-limb fat masses. Pancreatic and liver fat contents (log transformed) were significantly higher in the FPLD group (5.26 ± 1.5 vs. 4.08 ± 0.64, p = 0.034 and 0.77 ± 0.50 vs. 0.41 ± 0.18, p = 0.056, respectively). Pancreatic fat was inversely related to the DI (r = − 0.53, p = 0.027) and HDL-cholesterol (r = − 0.63, p = 0.003) and directly related to WHR (r = 0.60; p = 0.009), HbA1c (r = 0.58; p = 0.01) and serum triglyceride (r = 0.48, p = 0.034). Higher triglyceride and lower HDL-cholesterol levels were observed in the FPLD group.

**Conclusions:**

This study demonstrated for the first time that pancreatic fat deposition is increased in FPLD. Moreover, an inverse relationship was demonstrated between pancreatic fat and beta-cell function. The findings of this study may be consistent with the expandability hypothesis and the twin cycle hypothesis.

## Background

Lipodystrophies (LP) are a clinically heterogeneous group of genetic or acquired disorders characterized by a variable loss of subcutaneous adipose tissue [1]. Familial partial lipodystrophy (FPLD), which is currently the most common and well-described familial LP, is characterized by reduced subcutaneous (SC) fat deposition affecting the limbs and trunk, with a selective visceral lipodeposition and SC fat accumulation in the shoulder girdle, neck and face. FPLD patients exhibit marked insulin resistance with glucose intolerance or diabetes mellitus (DM), dyslipidemia, acanthosis nigricans and a high risk of cardiovascular (CV) disease [2–4]. The phenotype of FPLD most resembles the metabolic syndrome phenotype observed in the general population [4]. Therefore, a better understanding of this underexplored pathology can reveal important clues to decipher insulin resistance (IR) and its metabolic consequences.

Visceral adiposity and ectopic fat accumulations have been associated with FPLD [5–7]. These ectopic fat depositions might be related to organ dysfunction and adverse cardiometabolic effects. Our group described increased epicardial adipose tissue in a cohort of FPLD patients assessed by echocardiography [8]. Lipid accumulations in the liver and muscles were observed in mouse models of inherited LP [9]. Currently, an area of special interest is the possibility of pancreatic fat deposition in FPLD patients, a human model of insulin resistance [9, 10]. Chronic exposure of the pancreatic islets to non-esterified fatty acids is considered a potential primary cause of beta-cell dysfunction [10]. Indeed, recent evidence suggests that pancreatic lipid content may contribute to beta-cell dysfunction and possibly to the subsequent development of type 2 diabetes in susceptible individuals [11].

The most accurate measurements of body fat deposits are obtained using radiologic methods, namely, dual-energy X-ray absorptiometry (DXA) and magnetic resonance imaging (MRI), and techniques such as proton spectroscopy (H-MRS) and Dixon-based fat quantification (MRI-DIXON), especially for the determination of hepatic and pancreatic fat content [12–15]. These techniques have been validated against the direct determination of triglyceride content in human liver biopsies.

Currently, to the best of our knowledge, no data are available on the evaluation of fat deposition in the pancreas of FPLD patients. Furthermore, the relationship between pancreatic and hepatic fat deposition and beta-cell function has not been well studied. The aims of this study were to evaluate pancreatic and hepatic fat content and their correlation with beta-cell function in FPLD patients.

## Methods

### Subjects

Eleven women with FPLD from the outpatient clinics of Serviço de Metabologia do Instituto Estadual de Diabetes e Endocrinologia do Rio de Janeiro (Rio de Janeiro, Brazil) were included. The diagnosis of FPLD was confirmed by the molecular analysis of the *LMNA* gene (ABI Prism 3100 Genetic Analyzer; Applied Biosystems, Foster City, CA, USA) performed by the Molecular Endocrinology Laboratory of Paulista Medical School. Eleven patients had a missense mutation in the *LMNA* gene: seven patients harbored the heterozygous variant p.R482W (c.1444C>T), in three patients, the mutation identified was p.R482Q (c.1445G>A), and one patient exhibited a novel heterozygous variant in exon 8 (p.N466D (c.1396A>G) described previously [7]. The patients belonged to six different families.

A control group of 8 healthy women was carefully and sequentially selected to match the lipodystrophic group according to body mass index (BMI) and age. All controls were healthy and had no previous medical conditions likely to influence the evaluation at the time of the study.

The exclusion criteria were as follows: pregnant or breast-feeding women; severe renal or hepatic diseases; depression or alcoholism; use of thiazolidinediones (TZD) in the last 6 months or current use of glucocorticoids; and recent significant weight loss (≥ 3 kg in the last 3 months).

The study protocol was approved by the local ethics committee.

### Anthropometrical examination

The following anthropometrical data were recorded in all participants: body weight (kg), height (m), waist circumference (cm), waist-to-hip ratio (WHR), and blood pressure (mmHg). The BMI was calculated as weight in kilograms divided by the square of height in meters (kg/m^2^). The waist circumference was determined at the midpoint between the lowest rib and the iliac crest. The WHR was defined as the ratio of waist girth to the largest circumference of the hips, measured at the greater trochanter.

### Laboratory evaluation

#### Biochemical analyses

Blood samples were collected between 06:30 and 08:00 a.m. after an overnight fast (12 h). Plasma glucose was determined using the glucose-oxidase method. The cholesterol content of the lipoprotein fractions and triglycerides were measured enzymatically. Plasma leptin and insulin concentrations were measured using a radioimmunoassay. Fasting, 30, 60 and 120 min post load glucose levels were determined after a 75-g oral glucose tolerance test (OGTT).

#### Beta-cell function parameters

IR was estimated using the homeostasis model assessment (HOMA–IR) using the following formula: IR = fasting insulin × fasting glucose/22.5. The insulinogenic index (II) was calculated as the increment of insulin above the fasting level at 30 min divided by the corresponding glucose increment [16]. Since insulin secretion is dependent on insulin sensitivity, we evaluated the beta-cell disposition index (DI), calculated as the product of the II and the Matsuda index of insulin sensitivity (II × 1/insulin) [17, 18].

### Body fat analysis using DXA

A DXA scan (LUNAR PRODIGY ADVANCE software, version 9.5, LNR 41569 model; GE Medical Systems, Waukesha, WI, USA) was performed. The fat quantity and distribution were analyzed using the following variables: total fat (%), trunk fat (%), upper and lower limb fat (%), fat mass (g), central fat (g), and peripheral fat (g). The central-to-peripheral fat ratio or the fat mass ratio (FMR) was used to investigate body fat distribution, as previously described [5, 6, 19].

### Pancreas and liver fat deposition evaluated by MRI-DIXON

Subjects underwent imaging examinations of the liver and pancreas in the supine position via a 1.5 T Magnetom Avanto MR unit (Siemens Healthineers, Erlangen, Germany). After positioning the abdomen in a phased array coil, axial images were acquired from the level of the liver and pancreas using the two-point Dixon sequence as part of the routine clinical MRI protocol of the upper abdomen (not included for analysis in this study). The entire procedure, including positioning and scanning, was completed within 20 min. After completion of the automated reconstruction of the two-point Dixon images, the image sets of “fat only” and “water only” maps were transferred to a picture archiving and communication system (Carestream, Haifa, Israel) for analysis. The signal intensities in the images were calculated with operator-defined regions of interest (ROIs) at the same location in both fat and water images. A total of three ROIs per organ (liver and pancreas) were measured and averaged by a single investigator with care given to the placement of the ROIs to avoid confounding anatomy (e.g., large blood vessels). Fat/water ratios were calculated on a pixel-by-pixel basis by dividing the signal intensity calculated from the fat image by the signal intensity calculated from the water image (FF% = [F]/[W + F]). Decomposing fat and water signals to discriminate between fat and water protons based on their resonant frequency difference was first introduced by Dixon [15]. This method uses two acquisitions with a delay between the radiofrequency (RF) and gradient echoes such that the phase shift between the water and fat is either 0 or 90° (in-phase and out-of-phase, respectively). Separate water and fat images can be obtained by adding and subtracting the in-phase and out-of-phase images. The pulse sequence parameters were as follows: TR/TE in-phase 7.5/4.8 ms, TR/TE out-of-phase 7.5/2.4 ms, slice thickness 3 mm, matrix = 320 × 166, NEX = 1, and total scan time = 20 s.

### Statistical analysis

Statistical analysis was performed using GraphPad InStat 3.00 for Windows 95 (GraphPad So ware, San Diego, CA, USA). Parametric data are shown as mean ± standard deviation and non-parametric, such as median (range). An unpaired *t*-test was used to compare parametric variables, and the Mann–Whitney test or unpaired t-test (Welch corrected) were used for non-parametric variables. The strength of the linear relationship between two continuous variables was evaluated using Pearson or Spearman’s Coefficient. The level of statistical significance was 5%. Logarithmic-transformed values were used only for the liver fat variable due to the great dispersion of values.

## Results

Eleven women with FPLD were evaluated in our study. Ten patients were diagnosed with type 2 DM and were taking metformin, and one had impaired fasting glucose. The duration of diabetes varied from 1 to 10 years. Patients belonged to 6 different families as described in previous reports [5–7]. Table 1 describes baseline characteristics of the study population. The FPLD and control groups did not differ significantly with respect to age, BMI and waist circumference. As expected, the FPLD group presented a higher WHR and FMR and lower total, truncal and limb fat masses. The biochemical variables included higher triglycerides and lower HDL-cholesterol levels. Leptin was remarkably lower in the FPLD (4.2, 2.4–29.0 ng/ml) than in the control group (19.2, 11.5–27.4 ng/ml, p = 0.028).

**Table 1:** Anthropometrical examination, body fat analysis by DXA, pancreas and liver fat content by MRI-DIXON and biochemical variables in women with familial partial lipodystrophy (FPLD) and control groups

	Control (n = 8)	FPLD (n = 11)	p
Age (years)	32.3 (28.2–59.6)	33.0 (19.4–53.8)	0.65
BMI (kg/m^2^)	24.4 ± 2.8	23.3 ± 3.4	0.43
Waist (cm)	80.3 ± 4.3	81.3 ± 8.6	0.77
Hip (cm)	100.1 ± 6.9	88.2 ± 4.6	< 0.001
WHR	0.80 ± 0.04	0.92 ± 0.07	0.001
Total fat (%)	38.1 (32.8–46.2)	16.2 (13.1–27.6)	< 0.01
Trunk fat^a^ (%)	38.6 (31.5–47.3)	20.9 (16.3–34.6)	0.0018
Limbs fat^a^ (%)	44.1 ± 4.4	13.2 ± 4.4	< 0.001
FMR	0.89 ± 0.06	1.85 ± 0.40	< 0.001
Pancreas fat^b^ (%)	4.08 ± 0.64	5.26 ± 1.5	0.034
Liver fat^b^ (log)	0.41 ± 0.18	0.77 ± 0.50	0.056
Leptin (ng/ml)	19.2 (11.5–27.4)	4.2 (2.4–29.0)	0.028
Glucose (mg/dl)	81.5 (73.0–94.0)	102.0 (76.0–168.0)	0.025
HbA1c (%)	5.05 ± 0.41	6.20 ± 0.92	0.007
AST	17.5 (12.0–28.0)	18.0 (13.0–69.0)	0.77
ALT	15.0 (9.0–29.0)	22.0 (9.0–115.0)	0.14
Total Cholesterol (mg/dl)	196.5 ± 40.3	191.5 ± 26.9	0.75
HDL-c (mg/dl)	60.8 ± 11.3	40.5 ± 6.8	< 0.001
TG (mg/dl)	116 (47–160)	203 (103–392)	0.01
Disposition index	0.24 (0.13–2.07)	0.08 (0.01–0.17)	< 0.001

Data are mean ± SD or median (interval)

*FPLD* familial partial lipodystrophy, *BMI* body mass index, *HbA1c* hemoglobin A1c, *WHR* waist-to-hip ratio, *FMR* fat mass ratio (trunk/limbs fat), *HDL* high density lipoprotein, *TG* triglycerides, *DI* disposition index, *MRI* magnetic resonance imaging

^a^Body fat estimated from dual energy X-ray absorptiometry

^b^Pancreas and liver fat measured by MRI Dixon method

**Fig. 1:**
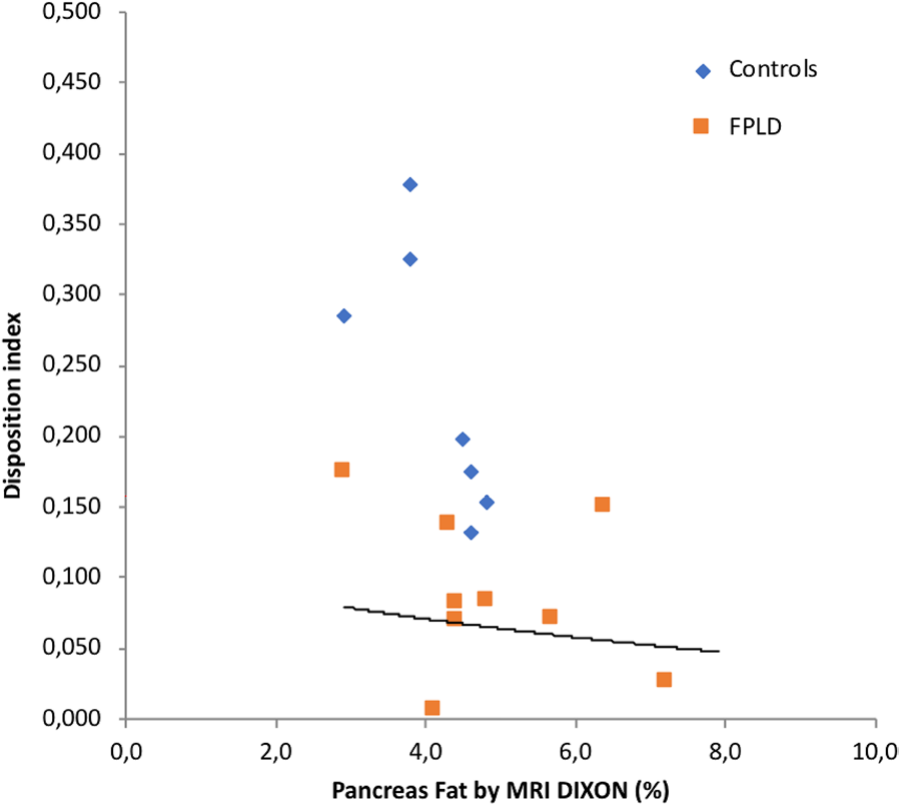
Correlation between disposition index (DI) and pancreatic fat measured by DIXON method (r = − 0.53, p = 0.027)

The main finding in this study was that the pancreatic fat content was significantly higher in the FPLD group than that in the control group (5.26 ± 1.50 vs. 4.08 ± 0.64, p = 0.034). In addition, the DI was significantly lower in the FPLD group [0.08 (0.01–0.17) vs 0.24 (0.13–2.07) p < 0.001]. Importantly, the pancreatic fat content was inversely related to the DI (r = − 0.53, p = 0.027; Fig. 1) and HDL-cholesterol level (r = − 0.63, p = 0.003) and directly related to WHR (r = 0.60; p = 0.009), HbA1c (r = 0.58; p = 0.01) and serum triglyceride (r = 0.48, p = 0.034). No correlation was found between pancreatic fat content and waist (r = 0.19; p = 0.43), total cholesterol (r = 0.21; p = 0.36), AST (r = 0.29; p = 0.21) or ALT (r = 0.42; p = 0.07).

Considering the great dispersion of values (ranging from 37.3 to 1.6%), log-transformed values were used to analyze the liver fat percentage. Liver fat was also increased in the FPLD group (0.77 ± 0.50 vs. 0.41 ± 0.18, p = 0.056).

## Discussion

For the first time in the literature, the current results show that patients with FPLD, have increased pancreatic fat as measured using the MRI-DIXON method. Similarly, in these patients, the pancreatic fat was inversely correlated with beta-cell function, suggesting that the mechanisms of beta-cell failure and diabetes may be associated with lipotoxicity [10–12].

As previously described, non-alcoholic fatty liver disease (NAFLD) is a commonly associated feature observed with IR in FPLD patients [1–4]. In our registry, liver fat was increased in 8 of 11 patients of the FPLD group. Although the log-transformed values were used for statistical analysis, the median fat percentage was 11.7%, which was similar to the findings of a cohort of 23 FPLD patients recently described by Ajnuli et al. [20] (11.9 ± 6.3%).

Liver and pancreatic fat deposition is consistent with the “twin cycle hypothesis”, which suggests that progressive liver and pancreatic fat accumulation will lead to a self-reinforcing cycle resulting in beta-cell dysfunction [21]. Interestingly, Lim et al. [22] demonstrated that after 8 weeks of a very low-calorie diet, the reversal of type 2 diabetes and complete beta-cell function recovery were temporarily coincident with a marked reduction in liver and pancreatic fat.

The expandability hypothesis suggests that a relative incapacity of adipose tissue to expand, and therefore store lipids, would cause excess fat to be stored in non-adipose tissues such as the liver and muscle, causing IR and lipotoxicity [23]. Peripheral fat scarcity (i.e., leg fat) is a hallmark of partial lipodystrophy [1, 2, 4]. In a previous report, Ajnuli et al. [20] showed that lower leg fat mass was correlated with higher triglycerides in FLPD patients, suggesting a pathogenic link between higher triglyceride levels and the incapacity to deposit fat in the lower leg. Similarly, these authors found an FMR above 1.5 in a cohort of 23 adult females with FPLD, a finding that was similar to ours (FMR = 1.85 in the FPLD group). Thus, lower leg fat mass may be the main reason why excess ingested energy accumulates in ectopic tissues, including the pancreas, therefore leading to metabolic impairment.

Normal weight metabolically unhealthy (NWMU) people have recently been characterized by genetic, anthropometric and body composition methods [24–27]. These studies highlight that common genetic variants associated with indices of IR or fat distribution are associated with metabolic features [25] and lower adiposity and BMI [24, 27], such as that found in monogenic lipodystrophies [1, 20, 28]. In addition, Stefan et al. [26] studied a cohort of 981 subjects who were at risk for metabolic diseases using whole-body MRI. Among the normal-weight subjects, ~ 20% were NWMU individuals who exhibited a high prevalence of low percentage of SC leg fat mass. When NWMU subjects were compared to metabolically unhealthy overweight or obese subjects, a gradual increase in the prevalence of low leg fat mass was found (β = 0.99, p < 0.0001), while a stronger increase was observed in the prevalence of NAFLD (β = 1.21, p < 0.0001) [26]. Taken together, this suggests a lipodystrophic-like phenotype.

Some limitations of this study must be acknowledged. We performed pancreatic fat quantification using Dixon method. The fatty infiltration of pancreas is increased during the fifth to seventh decade, therefore the use of this non-invasive technique in a younger and age-matched population may lead to a more accurate quantification [11, 12].

Additionally, most of our patients were already diagnosed with diabetes and one was glucose intolerant. In this study the gold-standard method to investigate beta-cell function, the hyperglycemic clamp method, was substituted by OGTT, which is widely used as well in literature and provides valuable information of “residual” beta-cell function [11, 17, 18]. According to the results obtained, we were able to confirm that the typical insulin resistant state observed in the FPLD patient is related to increased pancreatic fat content, when compared to controls, independent of aging or BMI.

This study is crucial to understanding this model of human IR and type 2 diabetes risk. It is consistent with an integrative genetic analysis of 53 genomic loci that reported that peripheral fat scarcity, and therefore limited fat storage capacity, is implicated in IR, diabetes and CV risk [27]. Indeed, in a recent Mendelian randomization study, a polygenic risk score of 48 SNPs for increased WHR (adjusted for BMI) provided causal evidence of the association with a higher risk for diabetes and coronary heart disease [29]. Notably, for each 1-SD genetic increase in WHR adjusted for BMI, an OR increment of 1.77 [95% CI 1.57–2.00] and of 1.46 [95% CI 1.32–1.62] for diabetes and CHD, respectively, was found.

In conclusion, this study demonstrated a unique feature of FPLD, specifically, an increase in pancreatic fat deposition as well as its inverse correlation with beta-cell function. Together with its typical peripheral fat scarcity, this model of IR diabetes is consistent with new evidence linking common genetic variants to a frequent lipodystrophic-like phenotype of lower leg fat mass, ectopic fat deposition, diabetes and CV risk. The findings of this study may be consistent with the expandability hypothesis and the twin cycle hypothesis.

## Abbreviations

LP: lipodystrophy
FPLD: familial partial lipodystrophy
IR: insulin resistance
NAFLD: non-alcoholic fatty liver disease
CV: cardiovascular
WHR: waist-to-hip ratio
DXA: dual-energy X-ray absorptiometry
MRI: magnetic resonance imaging and techniques
MRI-DIXON: Dixon-based fat quantification
BMI: body mass index
OGTT: oral glucose tolerance test
II: insulinogenic index
DI: beta-cell disposition index
FMR: fat mass ratio
NWMU: normal weight metabolically unhealthy
